# Supplementary figures and images for: The Shigella flexneri effector IpaH1.4 facilitates RNF213 degradation and protects cytosolic bacteria against interferon-induced ubiquitylation
Source: eLife. 2025 Nov 28;13:RP102714. doi: 10.7554/eLife.102714 (PMC12662631; doi:10.7554/eLife.102714)

A

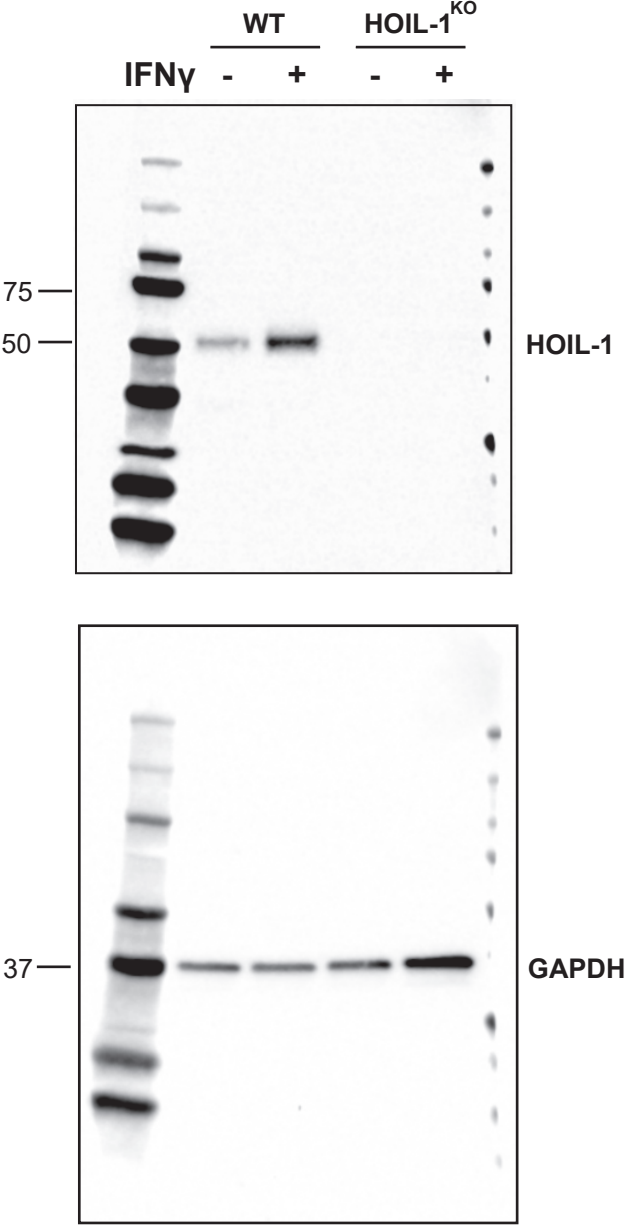

Figure 3A-HOIL Blot- Source Data 1

**A**

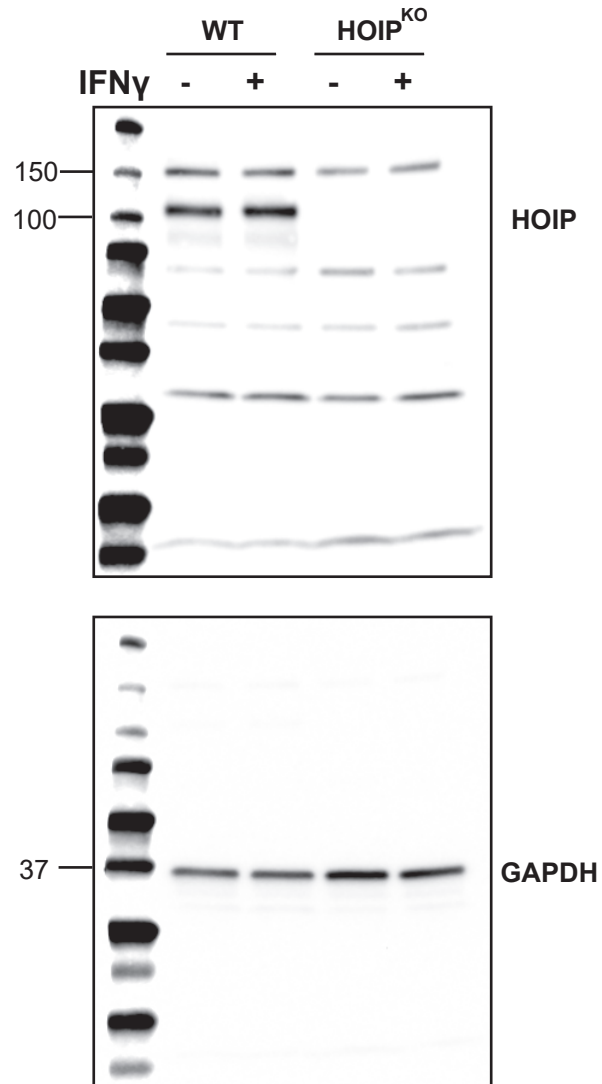

**Figure 3A-HOIP Blot- Source Data 1**

A

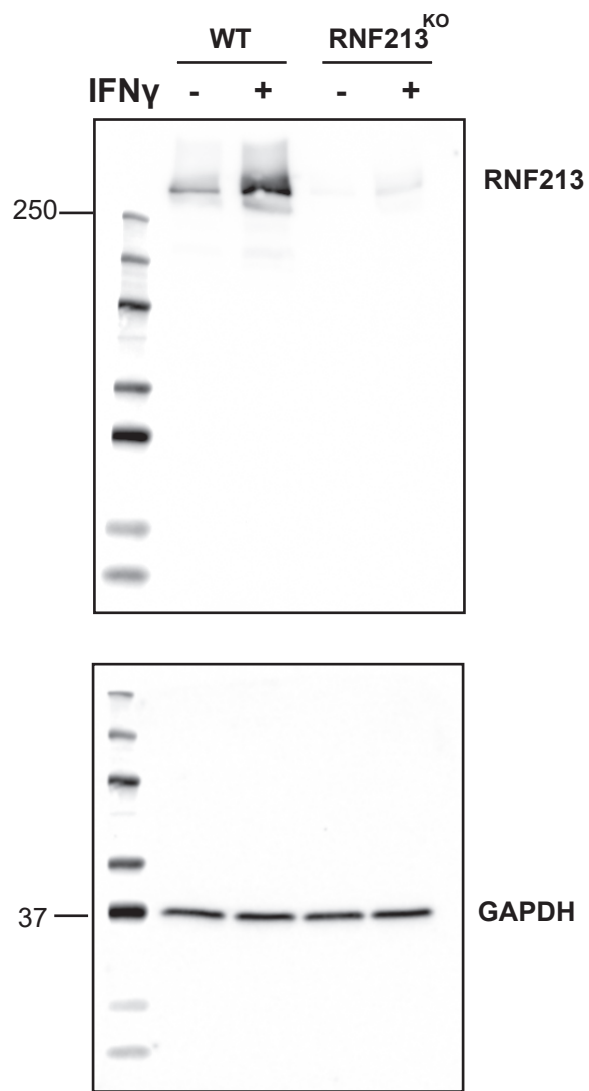

Figure 3A-RNF213 Blot- Source Data 1

Supplement: Figure 3—source data 1. [file elife-102714-fig3-data1.zip › Figure 3-Source-data-1/Figure_3A_Source Data 1.pdf]

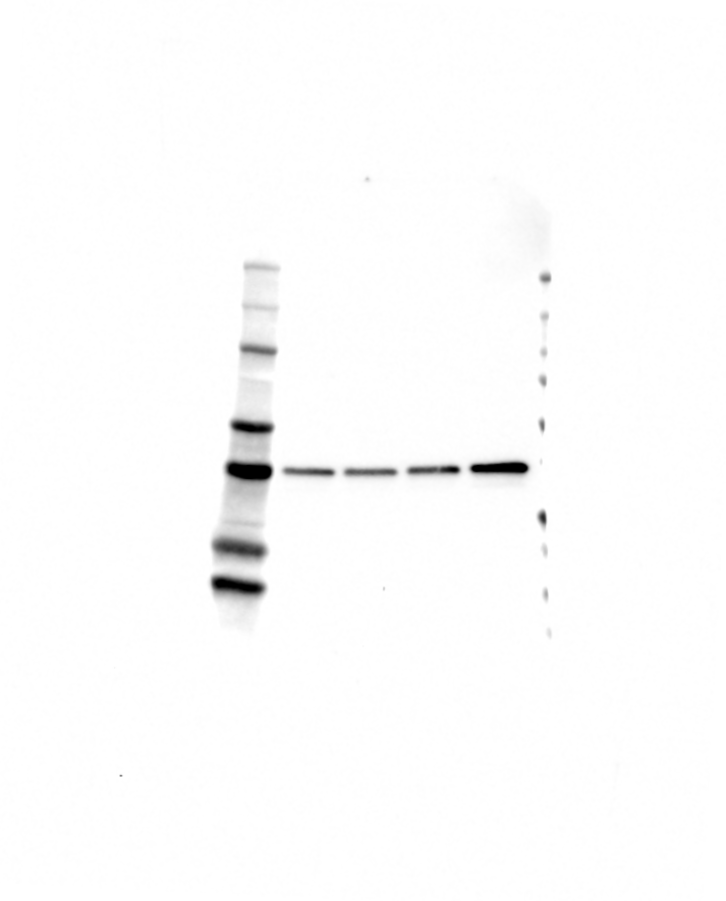

Supplement: Figure 3—source data 2. [file elife-102714-fig3-data2.zip › Figure 3-Source-data-2/Figure_3A_HOIL_Sourcedata2/WB_3A_HOIL-Panel_GAPDH+ladder.tif]

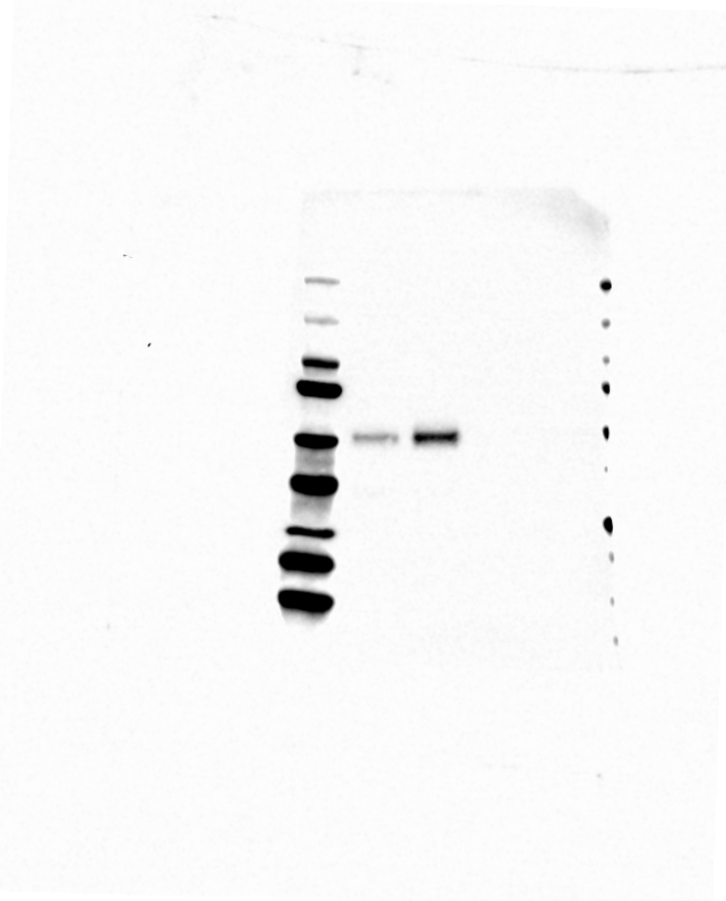

Supplement: Figure 3—source data 2. [file elife-102714-fig3-data2.zip › Figure 3-Source-data-2/Figure_3A_HOIL_Sourcedata2/WB_3A_HOIL-Panel_HOIL+ladder.tif]

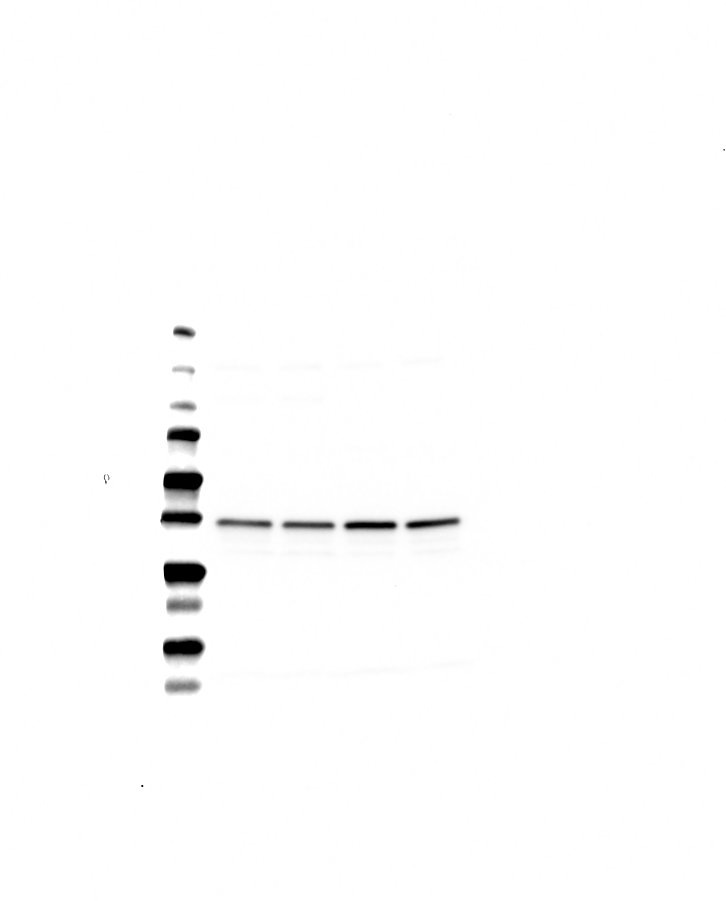

Supplement: Figure 3—source data 2. [file elife-102714-fig3-data2.zip › Figure 3-Source-data-2/Figure_3A_HOIP_Source-data-2/WB_3A_HOIP-Panel_GAPDH+Ladder.tif]

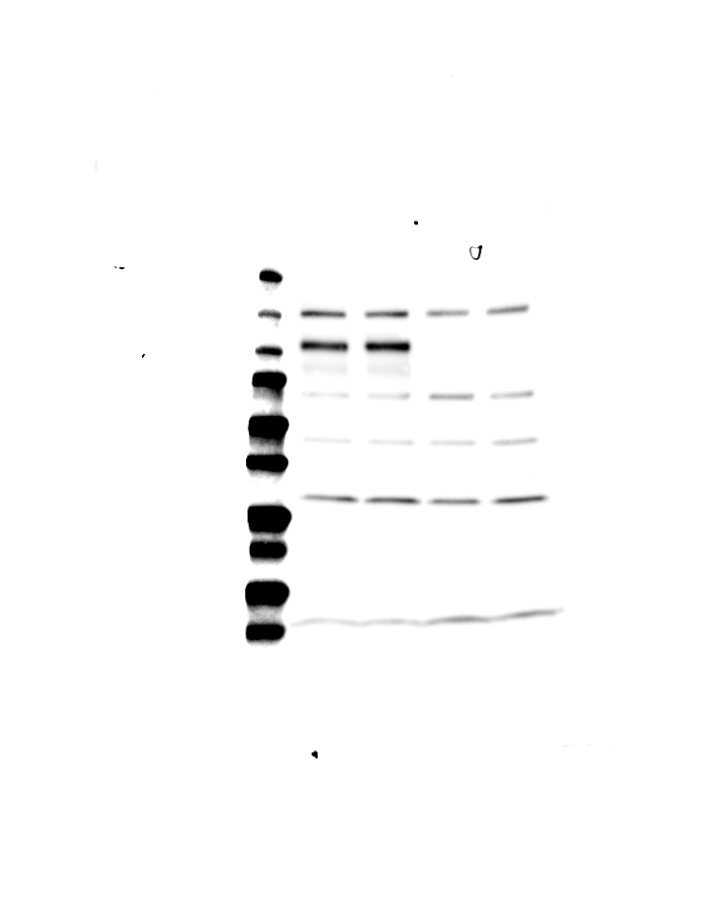

Supplement: Figure 3—source data 2. [file elife-102714-fig3-data2.zip › Figure 3-Source-data-2/Figure_3A_HOIP_Source-data-2/WB_3A_HOIP-Panel_HOIP+Ladder.tif]

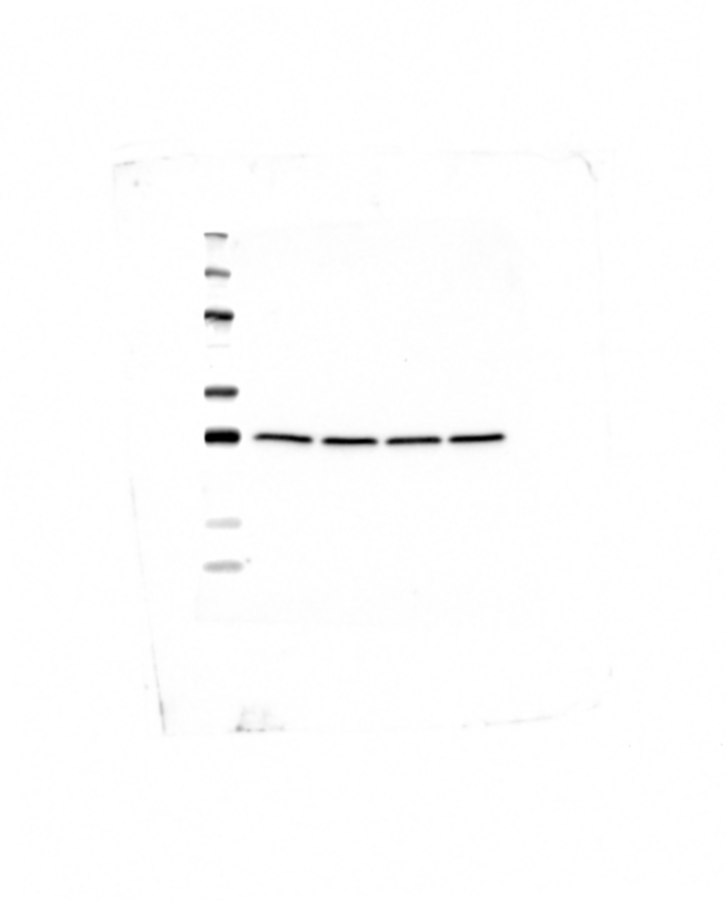

Supplement: Figure 3—source data 2. [file elife-102714-fig3-data2.zip › Figure 3-Source-data-2/Figure_3A_RNF213_Sourcedata2/WB_3A_RNF213-Panel_GAPDH+ladder.tif]

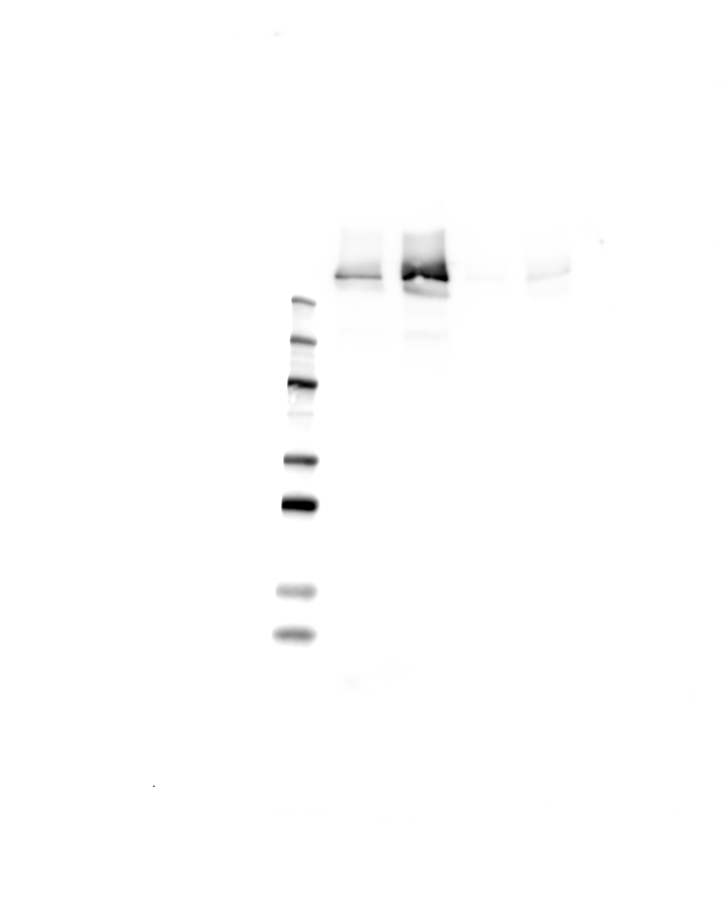

Supplement: Figure 3—source data 2. [file elife-102714-fig3-data2.zip › Figure 3-Source-data-2/Figure_3A_RNF213_Sourcedata2/WB_3A_RNF213-Panel_RNF213+ladder.tif]

**A**

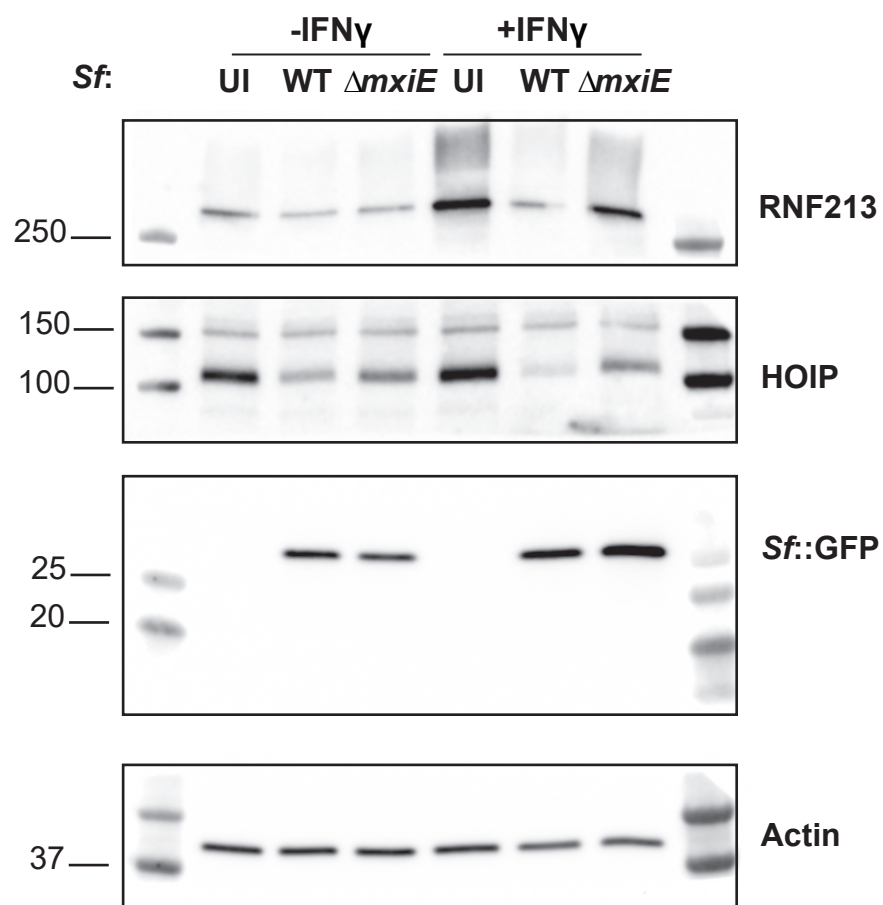

**Figure 4A- Source Data 1**

**B**

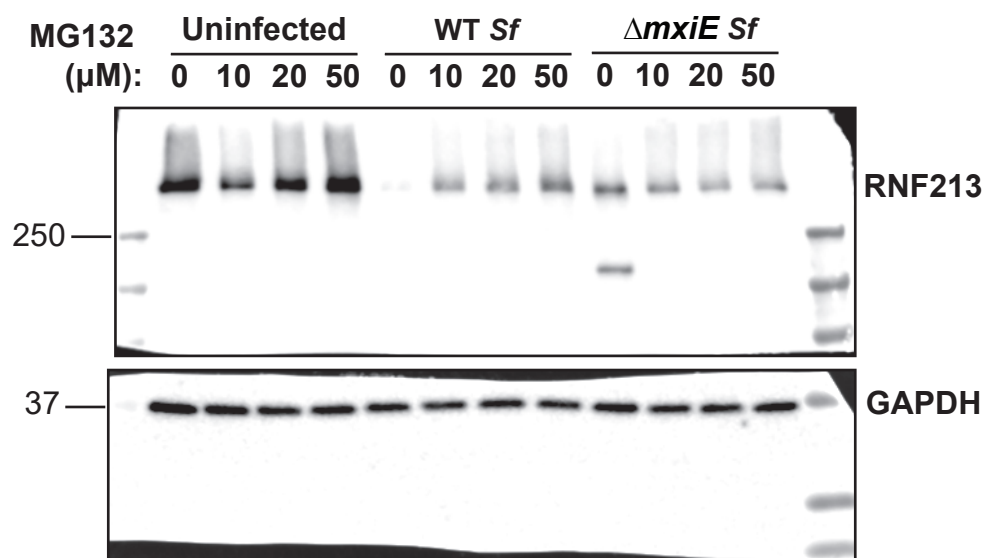

**Figure 4B- Source Data 1**

**C**

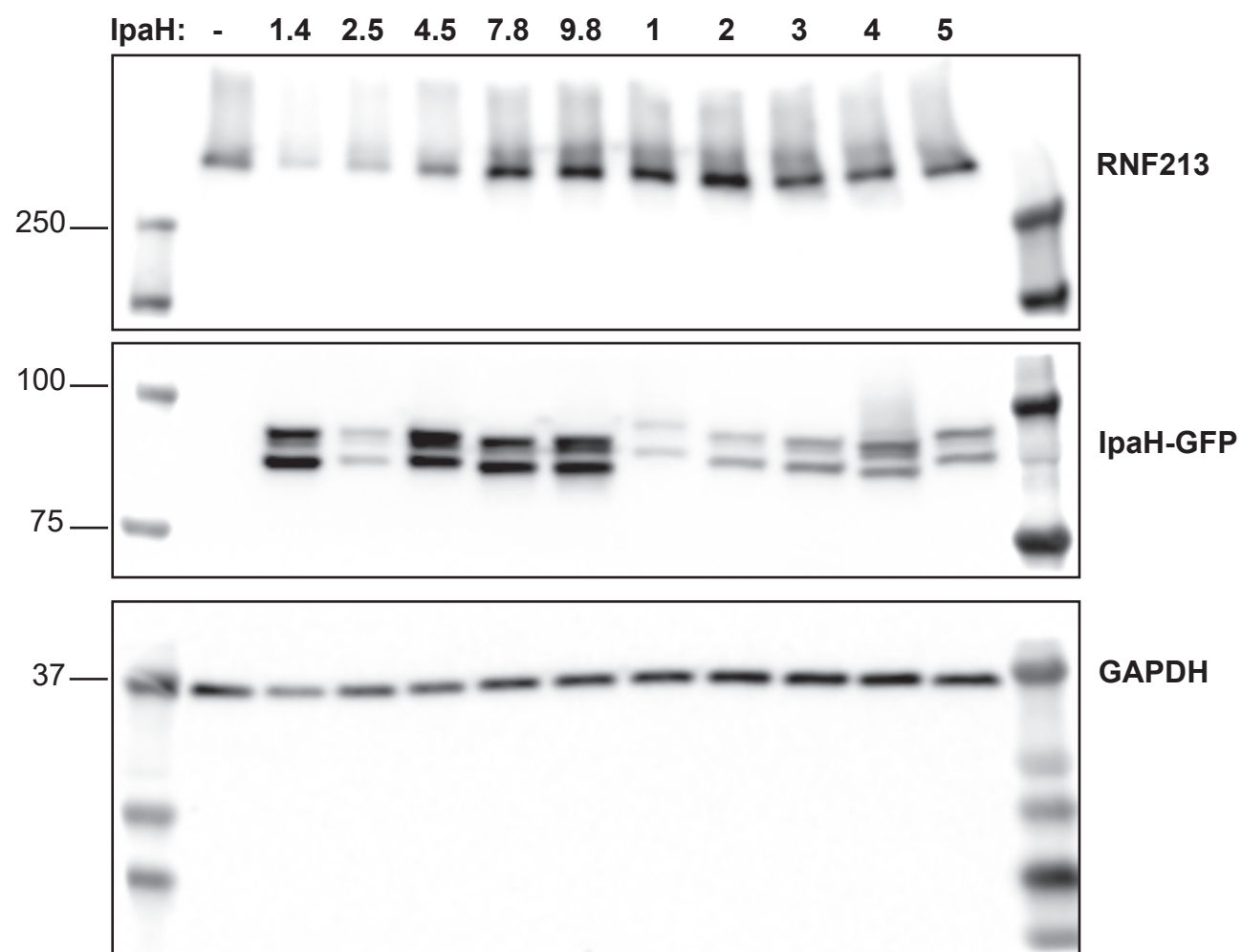

**Figure 4C- Source Data 1**

D

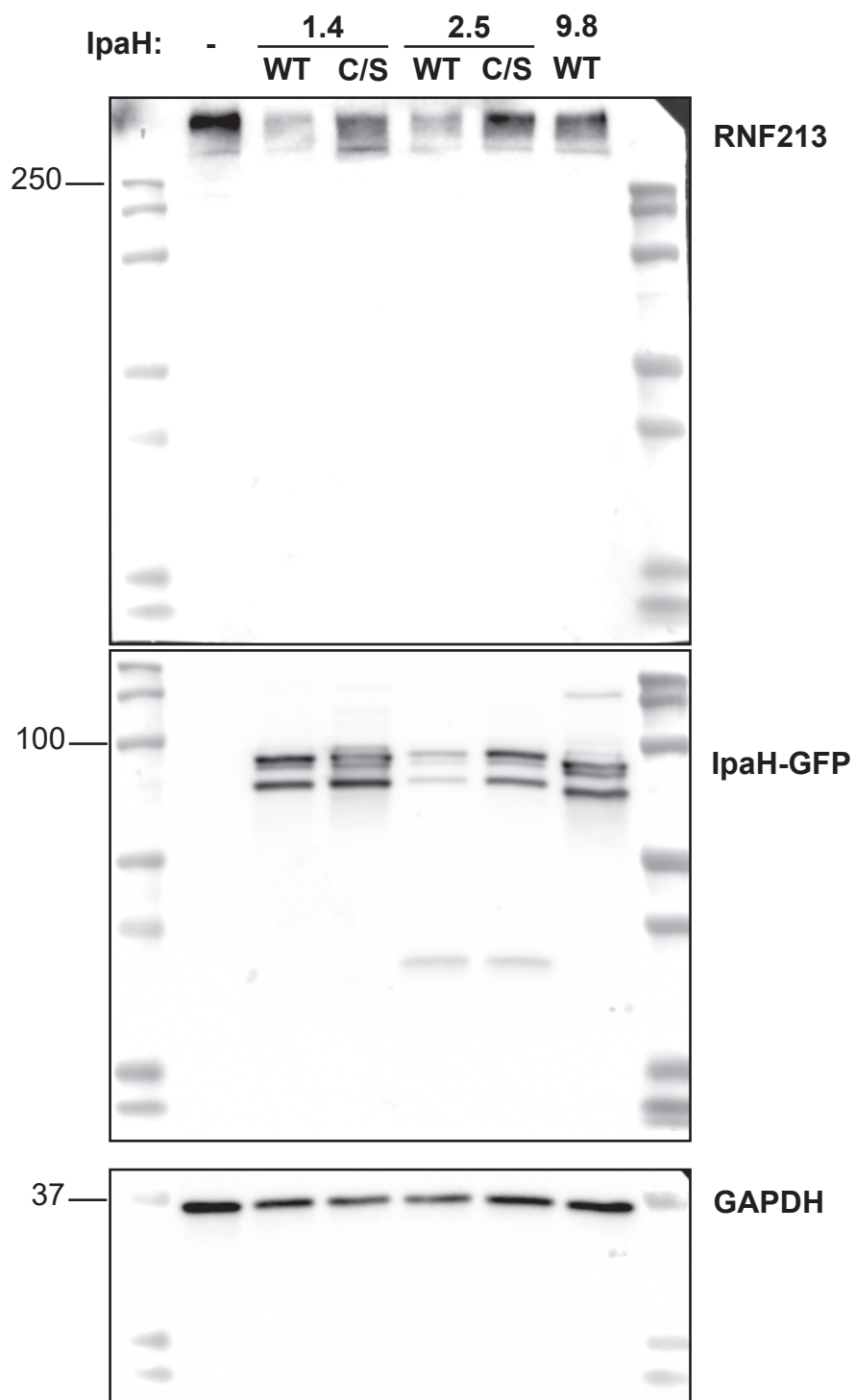

Figure 4D- Source Data 1

Supplement: Figure 4—source data 1. [file elife-102714-fig4-data1.zip › Figure 4-Source-data-1/Figure_4_Source Data 1.pdf]

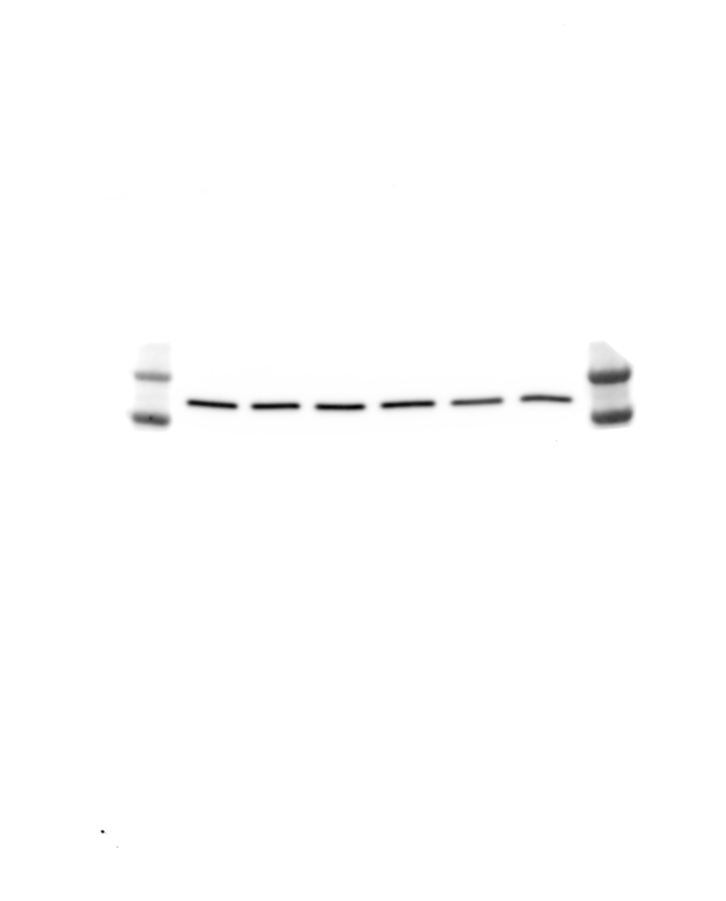

Supplement: Figure 4—source data 2. [file elife-102714-fig4-data2.zip › Figure 4-Source-data-2/Figure_4A_Sourcedata2/WB_4A_Actin+ladder.tif]

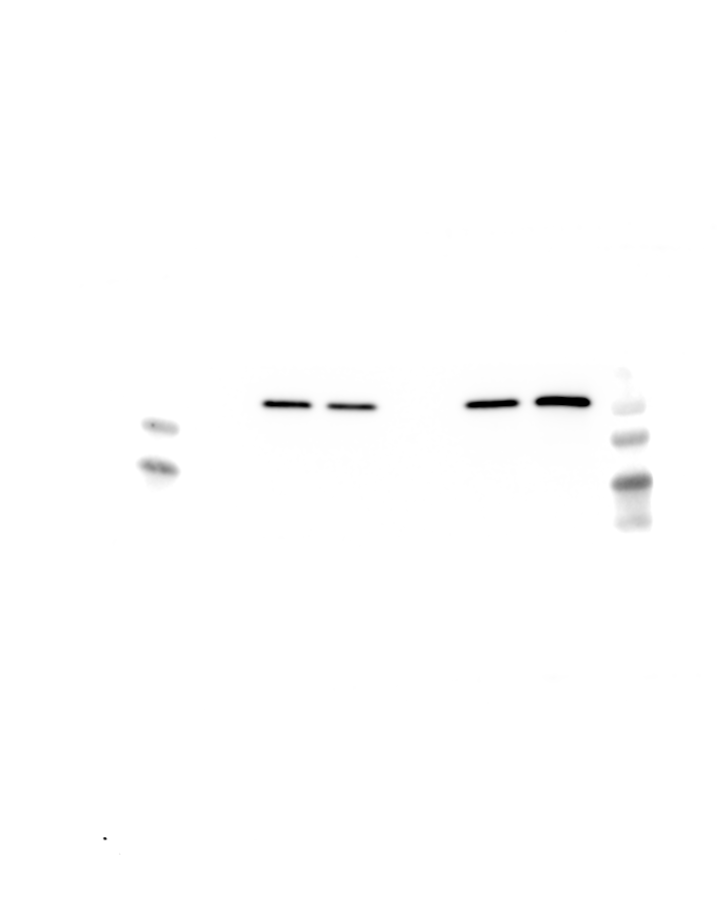

Supplement: Figure 4—source data 2. [file elife-102714-fig4-data2.zip › Figure 4-Source-data-2/Figure_4A_Sourcedata2/WB_4A_GFP+ladder.tif]

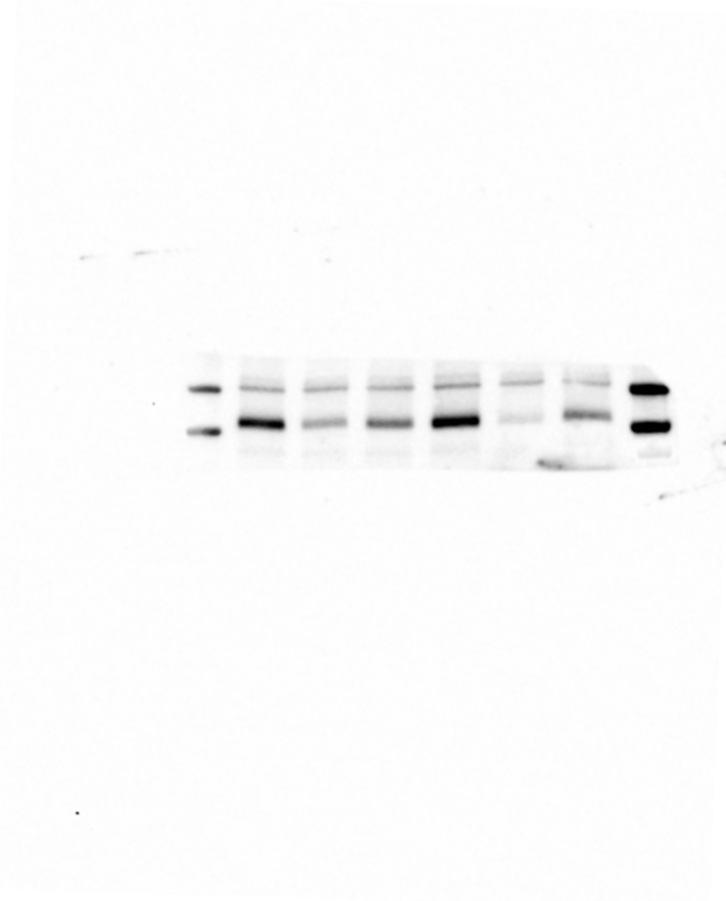

Supplement: Figure 4—source data 2. [file elife-102714-fig4-data2.zip › Figure 4-Source-data-2/Figure_4A_Sourcedata2/WB_4A_HOIP+ladder.tif]

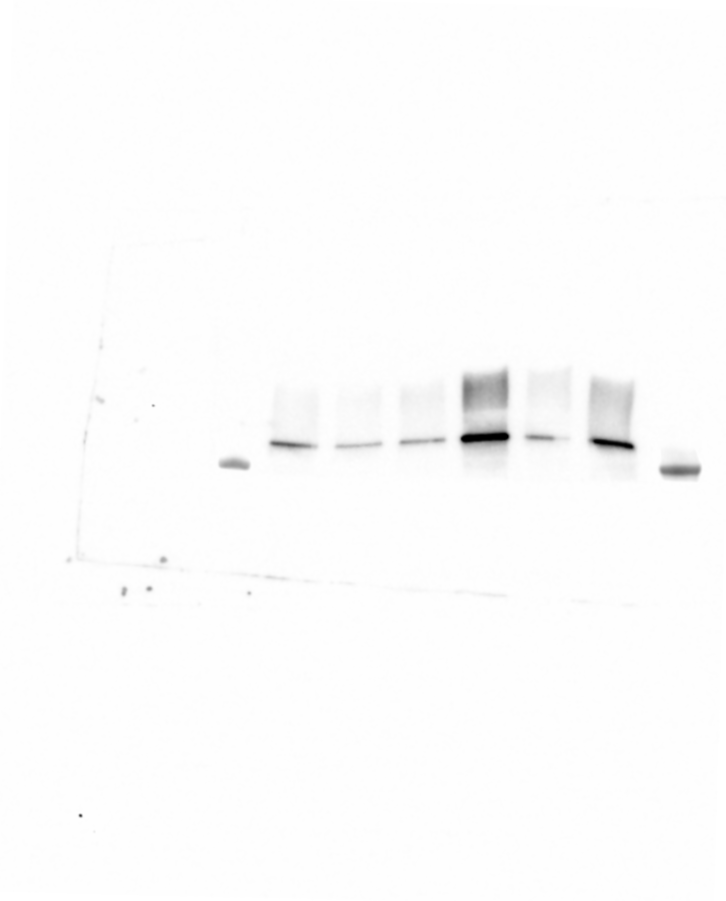

Supplement: Figure 4—source data 2. [file elife-102714-fig4-data2.zip › Figure 4-Source-data-2/Figure_4A_Sourcedata2/WB_4A_RNF213+ladder.tif]

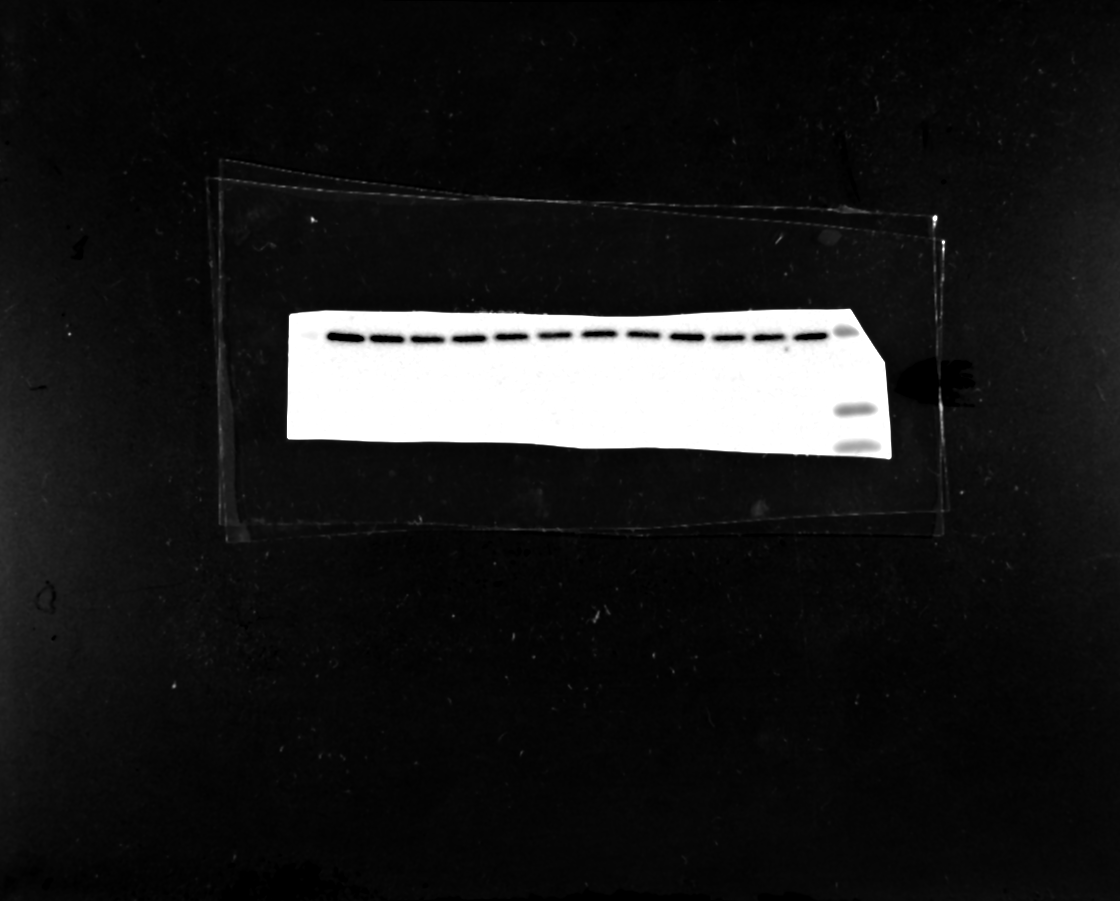

Supplement: Figure 4—source data 2. [file elife-102714-fig4-data2.zip › Figure 4-Source-data-2/Figure_4B_Sourcedata2/WB_4B_GAPDH+ladder.tif]

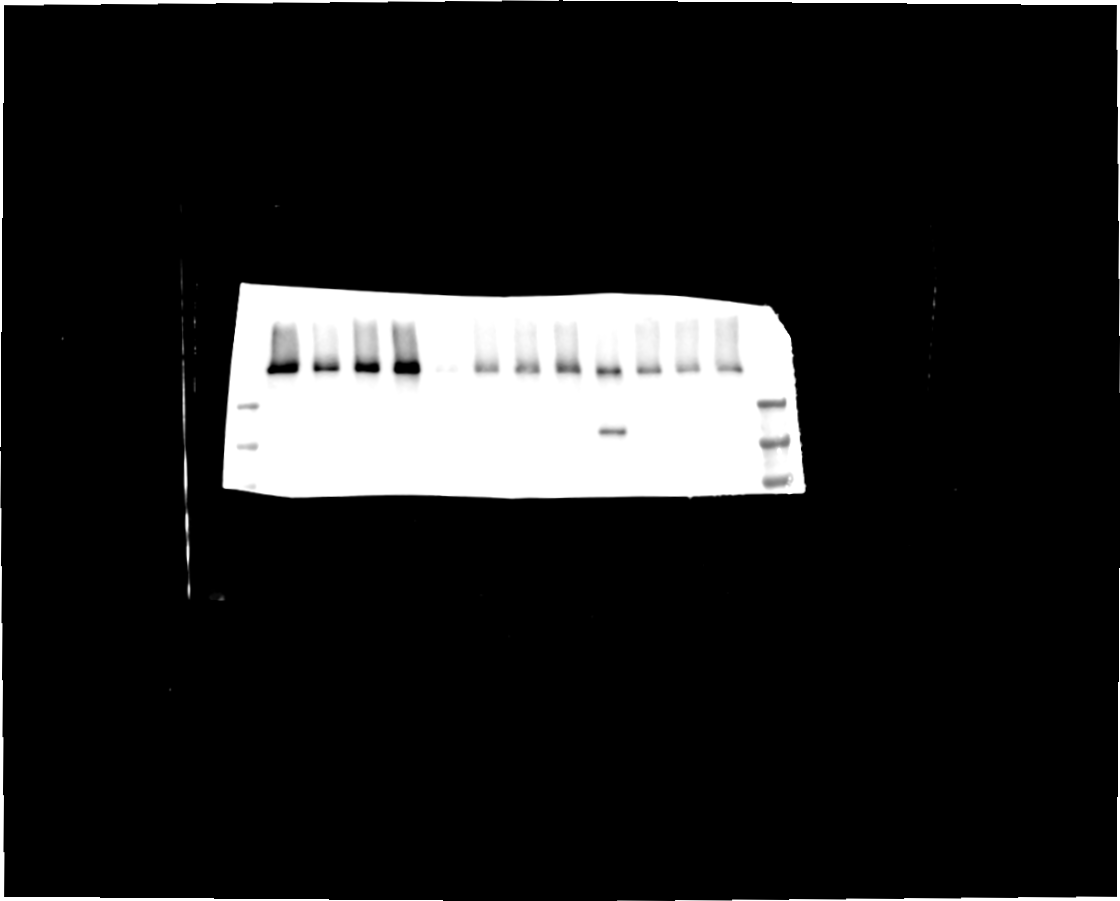

Supplement: Figure 4—source data 2. [file elife-102714-fig4-data2.zip › Figure 4-Source-data-2/Figure_4B_Sourcedata2/WB_4B_RNF213+ladder.tif]

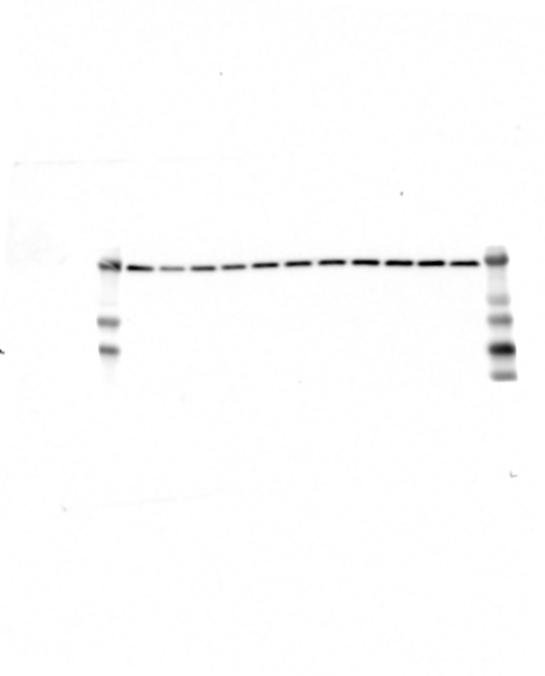

Supplement: Figure 4—source data 2. [file elife-102714-fig4-data2.zip › Figure 4-Source-data-2/Figure_4C_Sourcedata2/WB_4C_All-IpaH-Panel_GAPDH+ladder.tif]

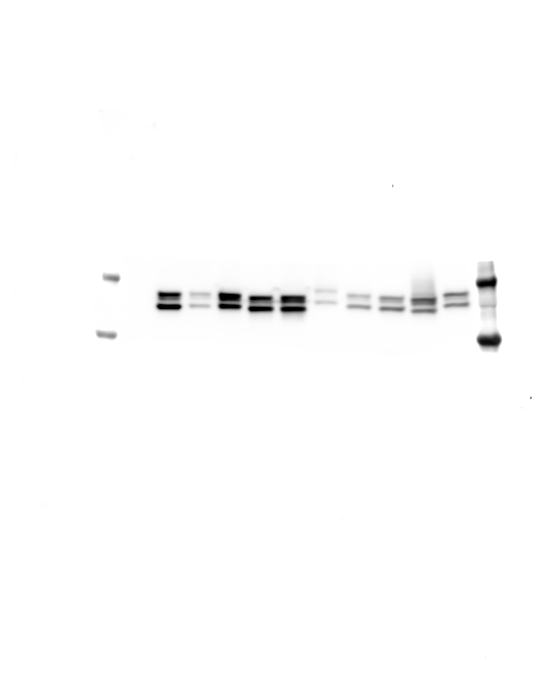

Supplement: Figure 4—source data 2. [file elife-102714-fig4-data2.zip › Figure 4-Source-data-2/Figure_4C_Sourcedata2/WB_4C_All-IpaH-Panel_GFP+ladder.tif]

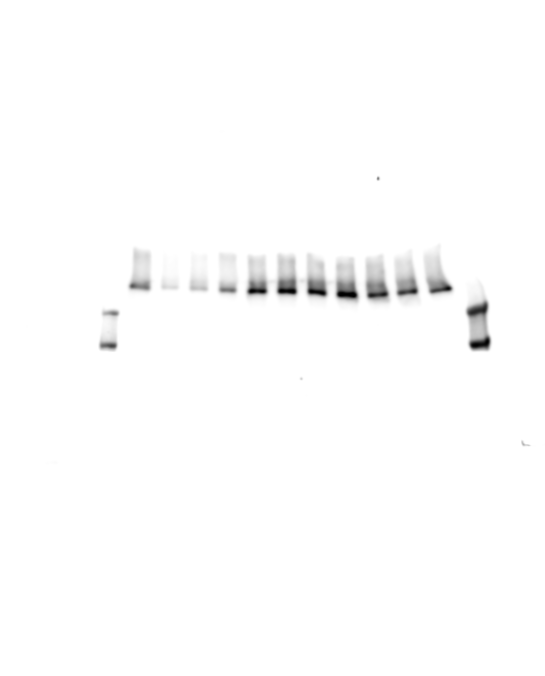

Supplement: Figure 4—source data 2. [file elife-102714-fig4-data2.zip › Figure 4-Source-data-2/Figure_4C_Sourcedata2/WB_4C_All-IpaH-Panel_RNF213+ladder.tif]

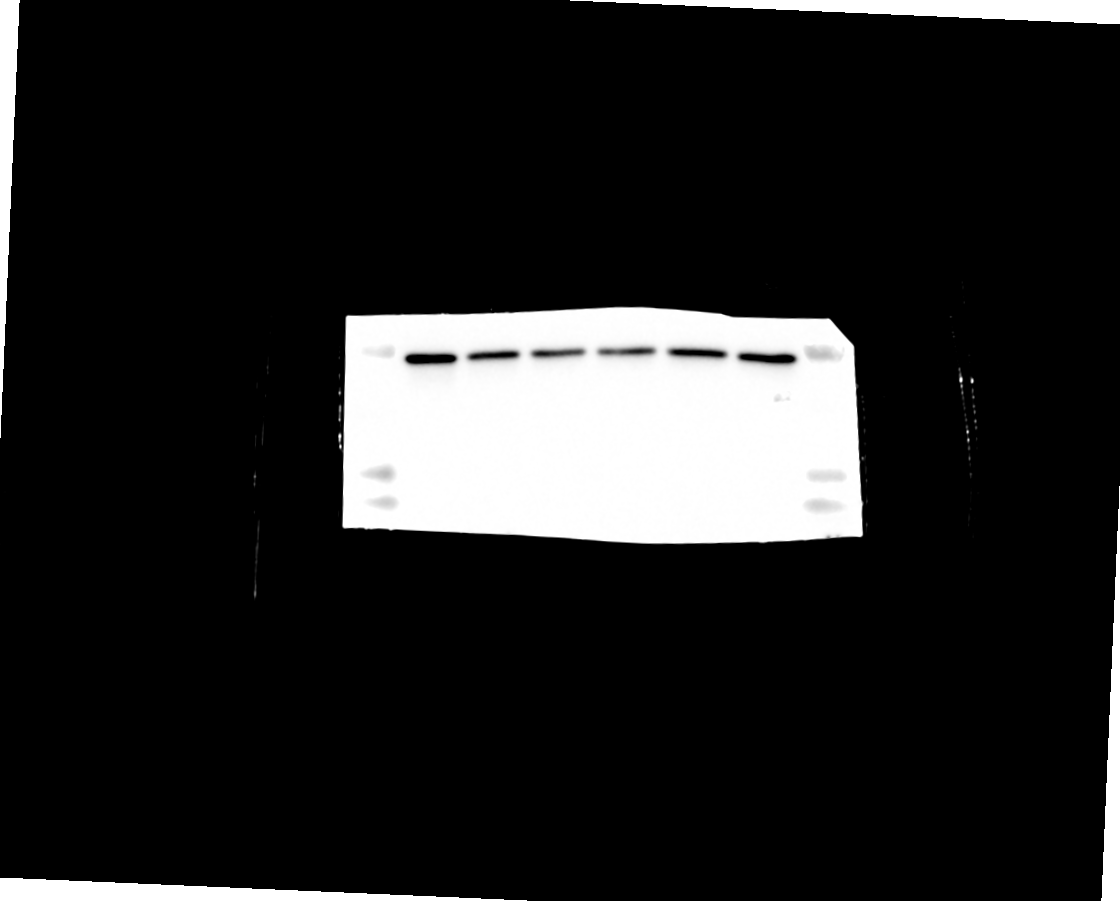

Supplement: Figure 4—source data 2. [file elife-102714-fig4-data2.zip › Figure 4-Source-data-2/Figure_4D_Sourcedata2/WB_4D_GAPDH+ladder.tif]

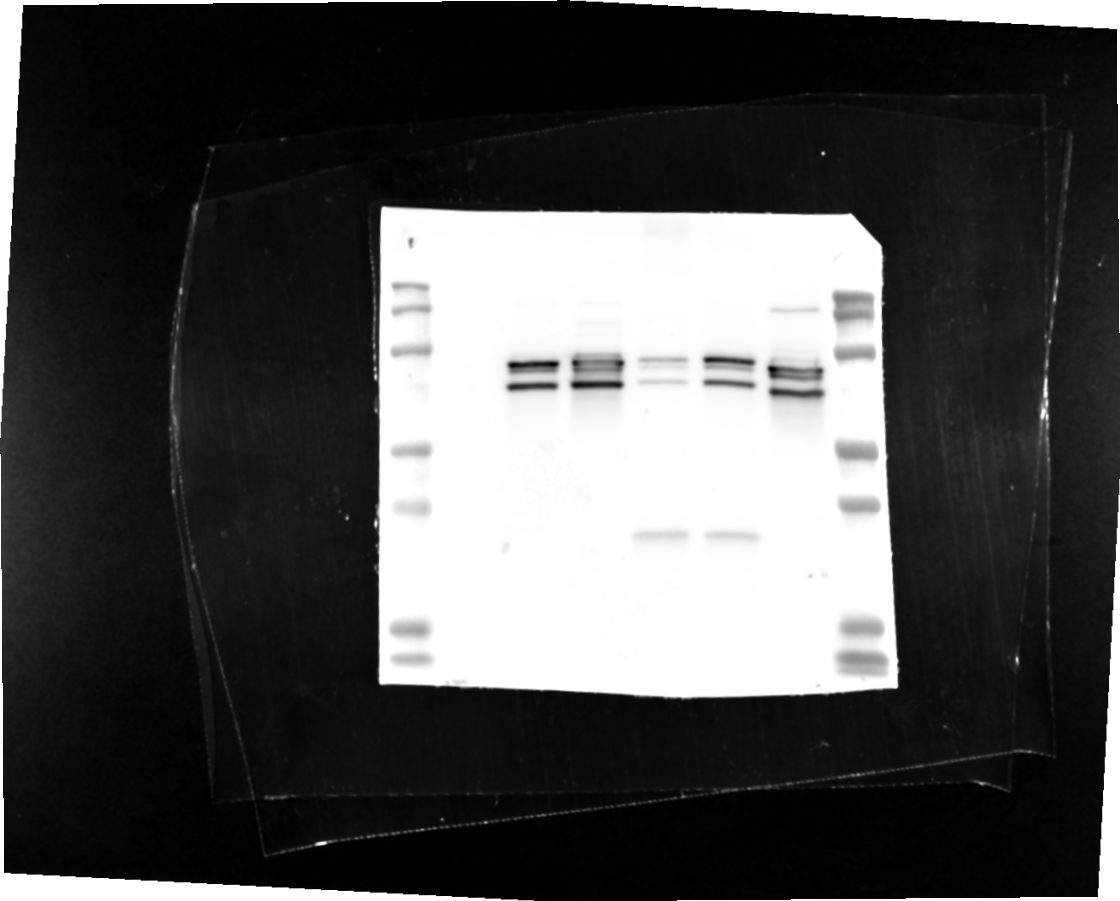

Supplement: Figure 4—source data 2. [file elife-102714-fig4-data2.zip › Figure 4-Source-data-2/Figure_4D_Sourcedata2/WB_4D_GFP+Ladder.tif]

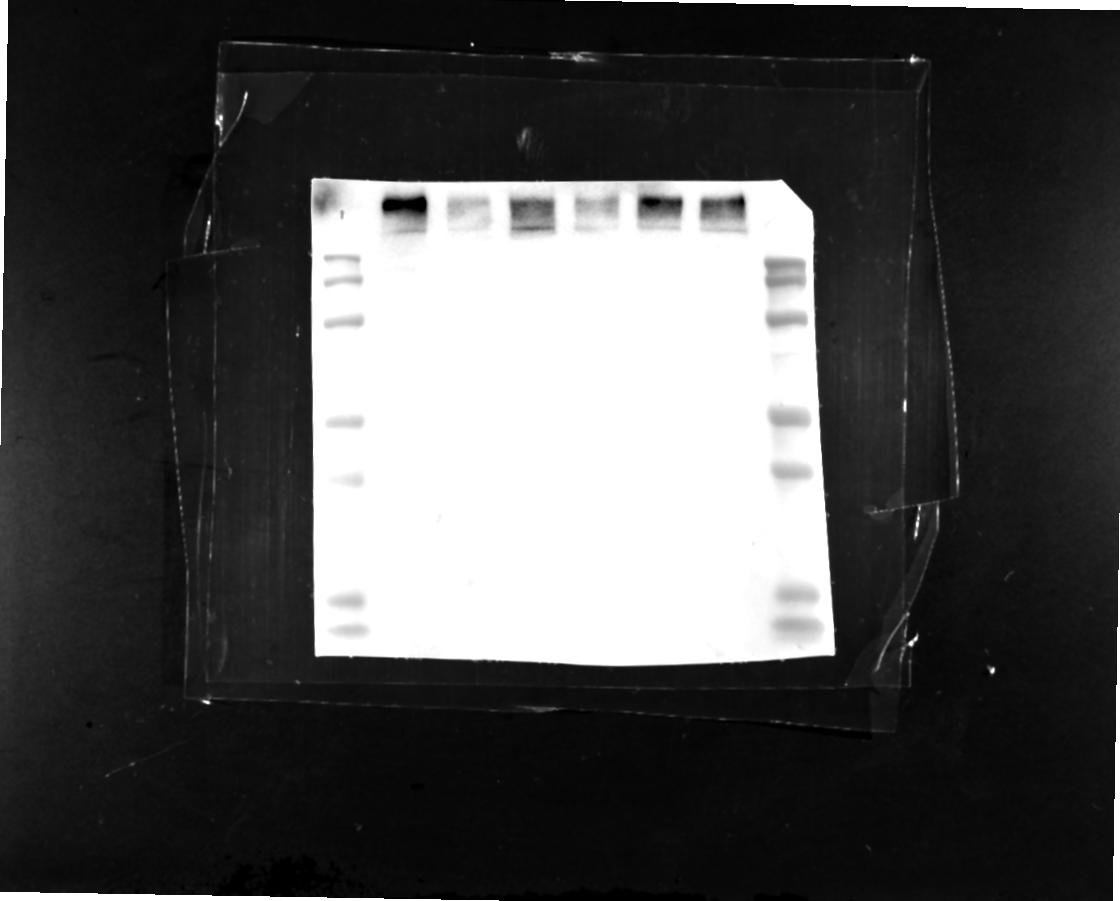

Supplement: Figure 4—source data 2. [file elife-102714-fig4-data2.zip › Figure 4-Source-data-2/Figure_4D_Sourcedata2/WB_4D_RNF213+ladder.tif]

A

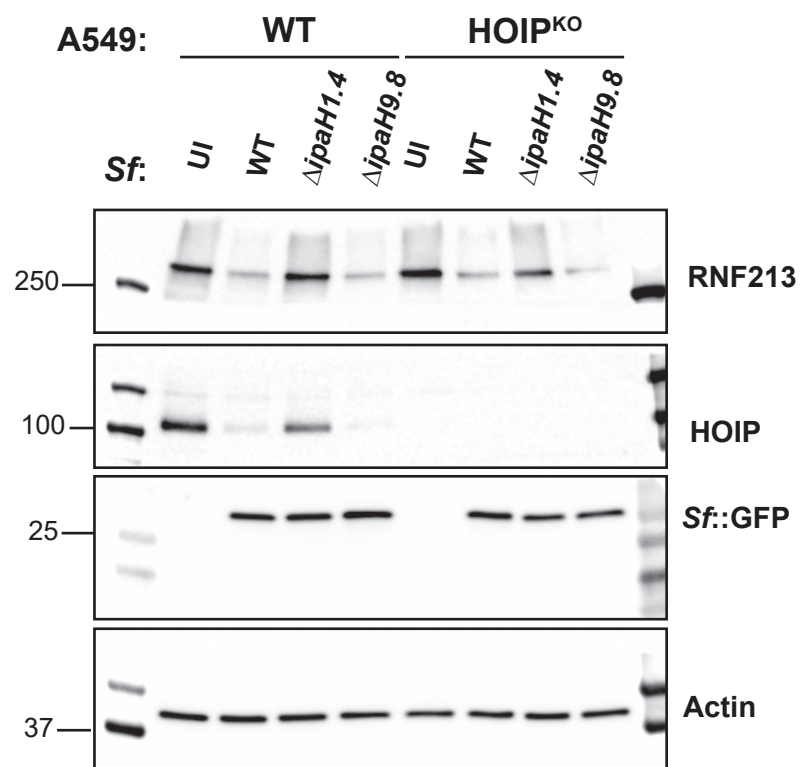

**Figure 5A- Source Data 1**

Supplement: Figure 5—source data 1. [file elife-102714-fig5-data1.zip › Figure 5-Source-data-1/Figure_5A_Source Data 1.pdf]

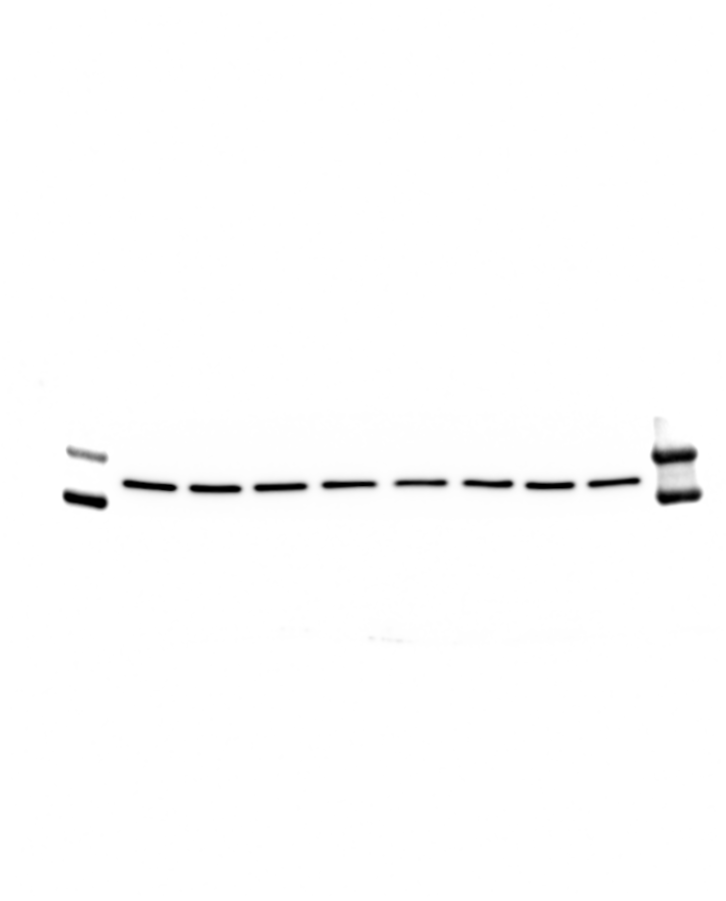

Supplement: Figure 5—source data 2. [file elife-102714-fig5-data2.zip › Figure 5_Source-data-2/WB_5A_actin+ladder.tif]

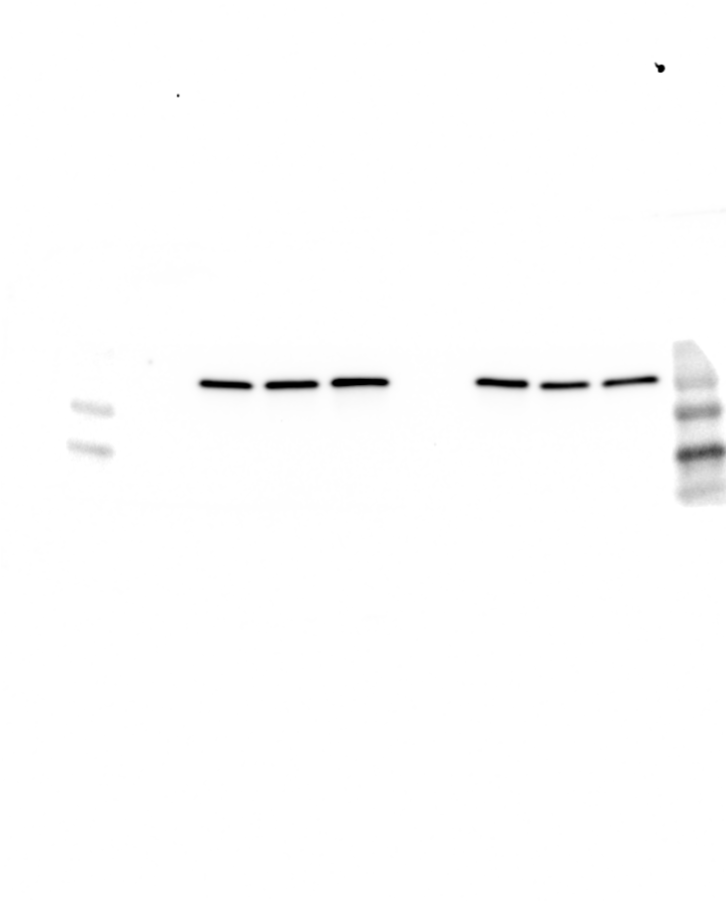

Supplement: Figure 5—source data 2. [file elife-102714-fig5-data2.zip › Figure 5_Source-data-2/WB_5A_GFP+ladder.tif]

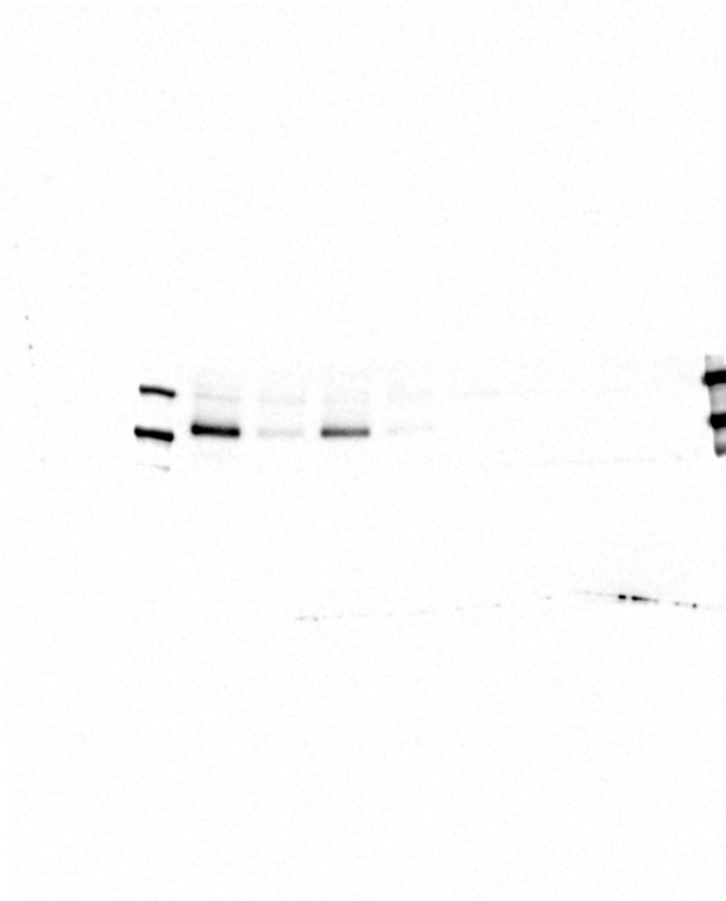

Supplement: Figure 5—source data 2. [file elife-102714-fig5-data2.zip › Figure 5_Source-data-2/WB_5A_HOIP+ladder.tif]

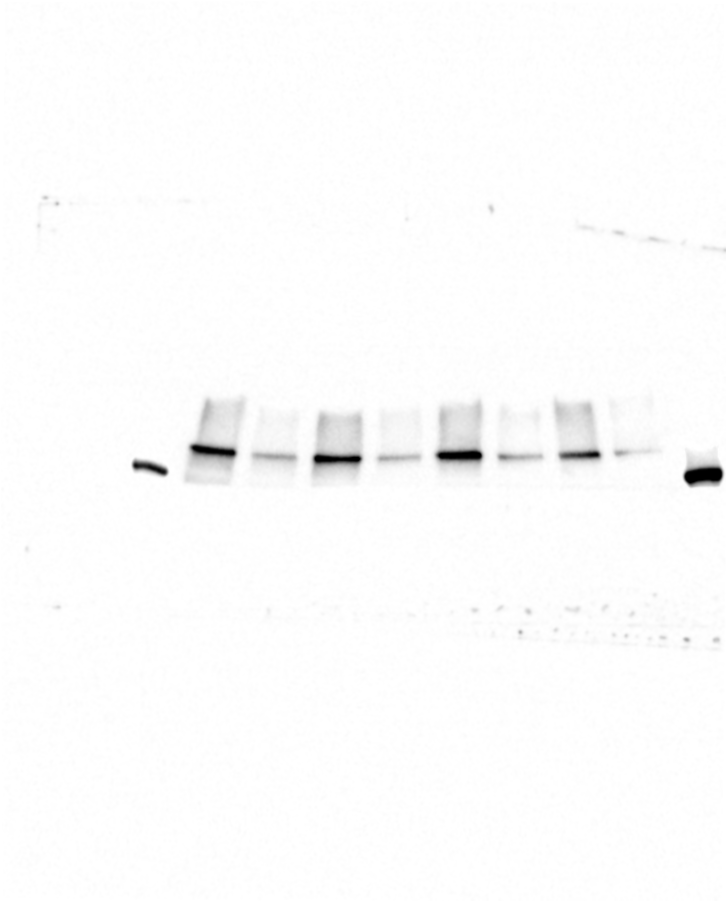

Supplement: Figure 5—source data 2. [file elife-102714-fig5-data2.zip › Figure 5_Source-data-2/WB_5A_RNF213+ladder.tif]

**B**

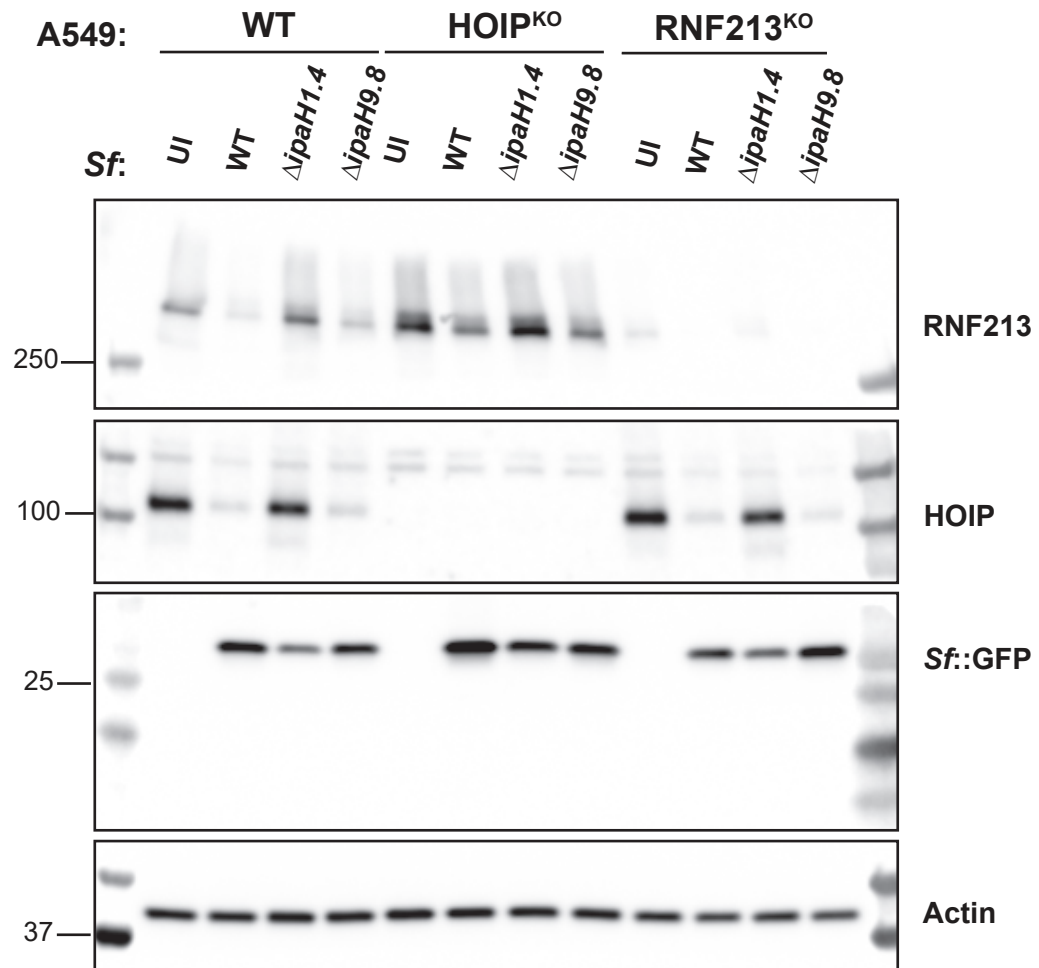

**Figure 5(Figure Supplement 1) - Source Data 1**

Supplement: Figure 5—figure supplement 1—source data 1. [file elife-102714-fig5-figsupp1-data1.zip › Figure 5_Figure Supplement 1-Source-data-1/Figure 5 - Figure Supplement 1_Source data 1.pdf]

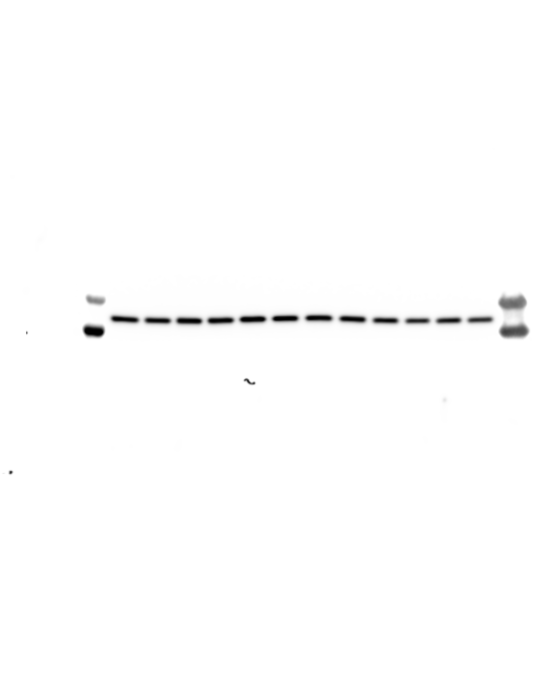

Supplement: Figure 5—figure supplement 1—source data 2. [file elife-102714-fig5-figsupp1-data2.zip › Figure 5_Figure Supplement 1-Source-data-2/WB_S3B_Actin+Ladder.tif]

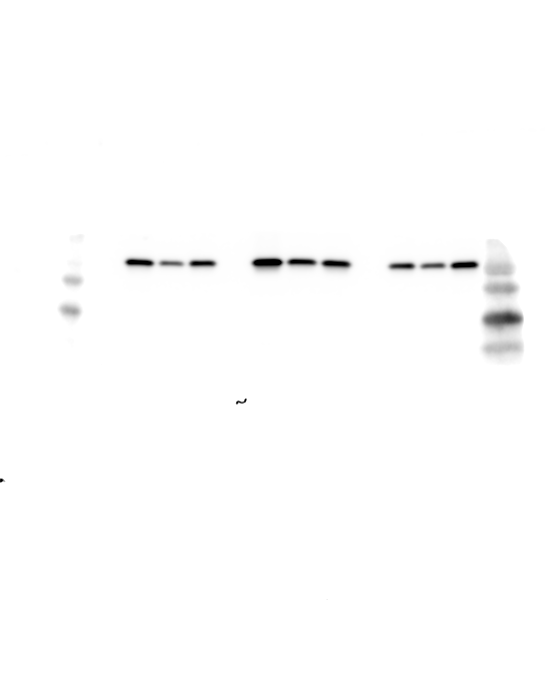

Supplement: Figure 5—figure supplement 1—source data 2. [file elife-102714-fig5-figsupp1-data2.zip › Figure 5_Figure Supplement 1-Source-data-2/WB_S3B_GFP+Ladder.tif]

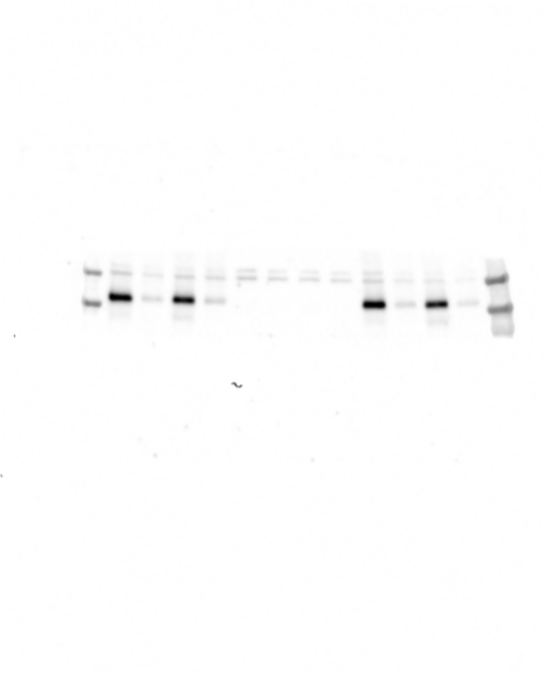

Supplement: Figure 5—figure supplement 1—source data 2. [file elife-102714-fig5-figsupp1-data2.zip › Figure 5_Figure Supplement 1-Source-data-2/WB_S3B_HOIP+Ladder.tif]

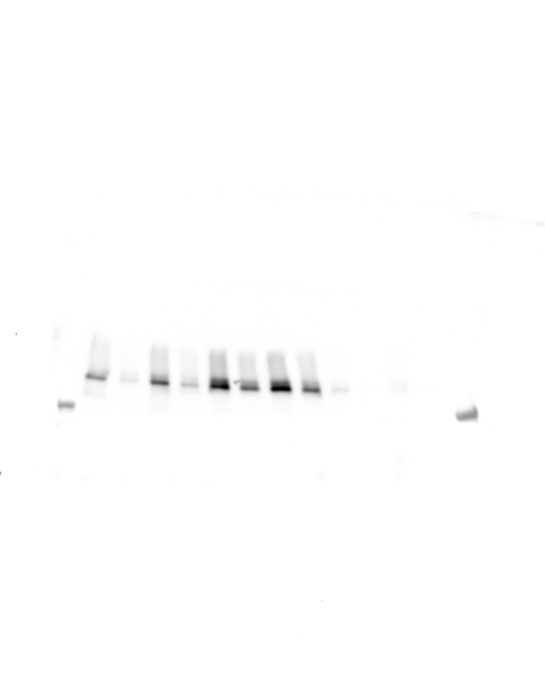

Supplement: Figure 5—figure supplement 1—source data 2. [file elife-102714-fig5-figsupp1-data2.zip › Figure 5_Figure Supplement 1-Source-data-2/WB_S3B_RNF213+Ladder.tif]

**A**

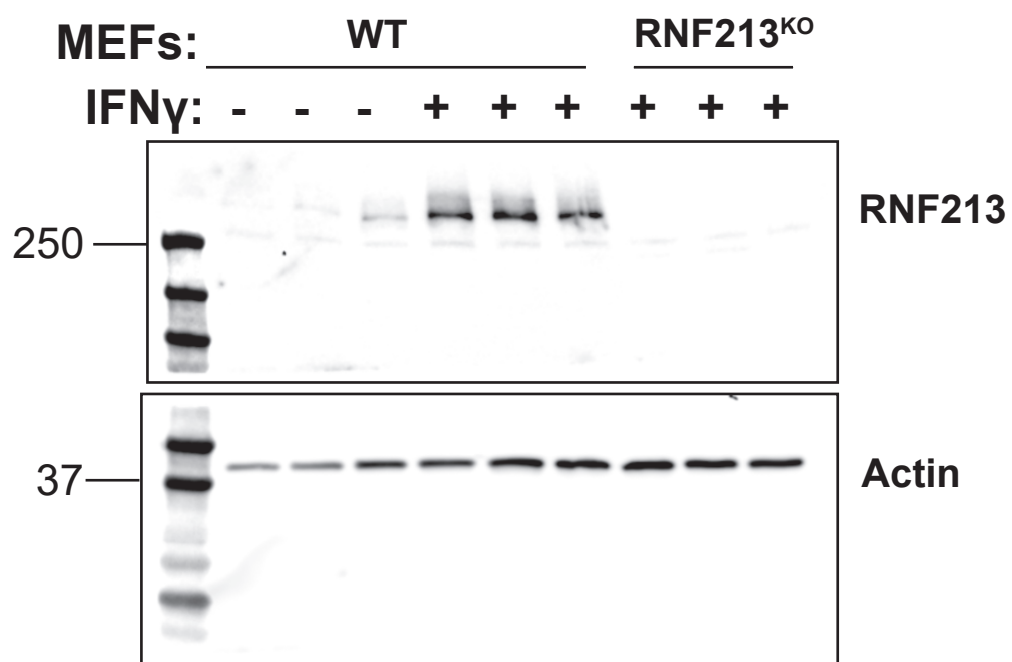

**Figure 5(Figure Supplement 2) - Source Data 1**

Supplement: Figure 5—figure supplement 2—source data 1. [file elife-102714-fig5-figsupp2-data1.zip › Figure 5_Figure Supplement 2-Source-data-1/Figure 5 - Figure Supplement 2_Source data 1.pdf]

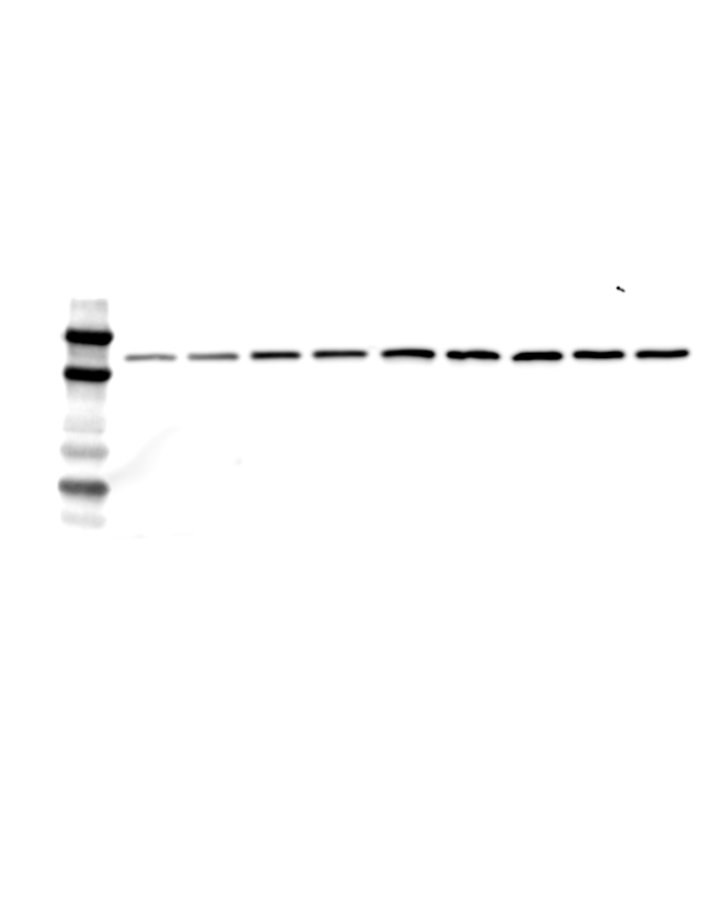

Supplement: Figure 5—figure supplement 2—source data 2. [file elife-102714-fig5-figsupp2-data2.zip › Figure 5_Figure Supplement 2-Source-data-2/WB_S4A_mActin+ladder.tif]

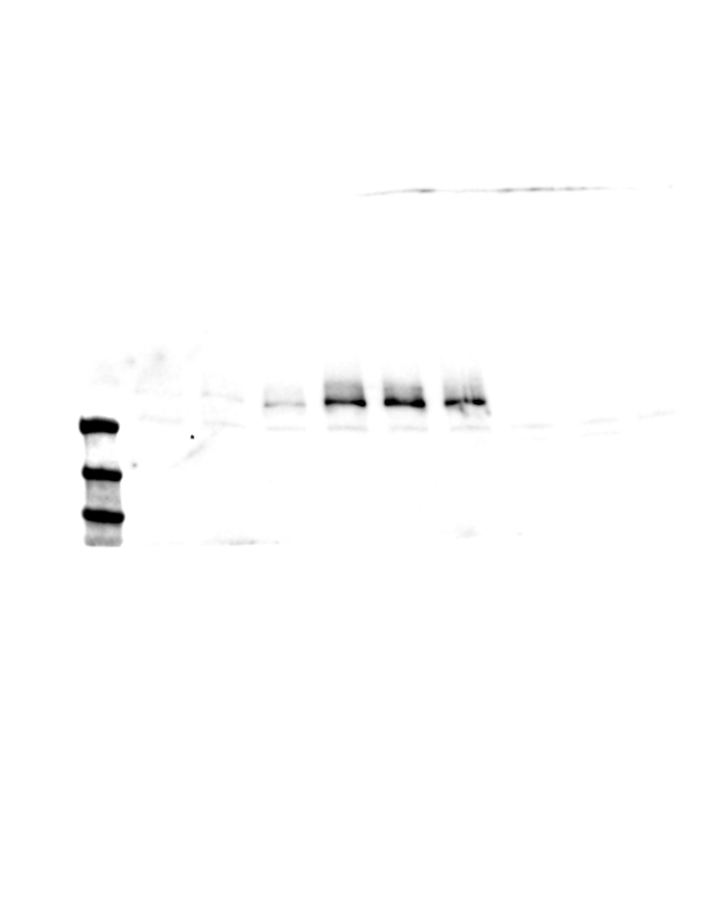

Supplement: Figure 5—figure supplement 2—source data 2. [file elife-102714-fig5-figsupp2-data2.zip › Figure 5_Figure Supplement 2-Source-data-2/WB_S4A_mRNF213-ab4073+Ladder.tif]
