# Supplementary material for: The Shigella flexneri effector IpaH1.4 facilitates RNF213 degradation and protects cytosolic bacteria against interferon-induced ubiquitylation
Source: eLife. 2025 Nov 28;13:RP102714. doi: 10.7554/eLife.102714 (PMC12662631; doi:10.7554/eLife.102714)
Supplement: Supplementary file 1. [file elife-102714-supp1.docx]

**Table 1: Internally Strep tagged Ubiquitin constructs**

| **Construct** | **Aminoacidic sequence (ORF)** |
| --- | --- |
| WT Ub | MQIFVKTLTGKTITLEVEPSDTIENVKAKIQDKEGIPPDQQRLIFAGKQLEDGSAWSHPQFEKSATLSDYNIQKESTLHLVLRLRGG |
| 7KR Ub | MQIFVRTLTGRTITLEVEPSDTIENVRARIQDREGIPPDQQRLIFAGRQLEDGSAWSHPQFEKSATLSDYNIQRESTLHLVLRLRGG |
| 6KR+K6 Ub | MQIFVKTLTGRTITLEVEPSDTIENVRARIQDREGIPPDQQRLIFAGRQLEDGSAWSHPQFEKSATLSDYNIQRESTLHLVLRLRGG* |
| 6KR+K11 Ub | MQIFVRTLTGKTITLEVEPSDTIENVRARIQDREGIPPDQQRLIFAGRQLEDGSAWSHPQFEKSATLSDYNIQRESTLHLVLRLRGG* |
| 6KR+K27 Ub | MQIFVRTLTGRTITLEVEPSDTIENVKARIQDREGIPPDQQRLIFAGRQLEDGSAWSHPQFEKSATLSDYNIQRESTLHLVLRLRGG* |
| 6KR+K29 Ub | MQIFVRTLTGRTITLEVEPSDTIENVRAKIQDREGIPPDQQRLIFAGRQLEDGSAWSHPQFEKSATLSDYNIQRESTLHLVLRLRGG* |
| 6KR+K33 Ub | MQIFVRTLTGRTITLEVEPSDTIENVRARIQDKEGIPPDQQRLIFAGRQLEDGSAWSHPQFEKSATLSDYNIQRESTLHLVLRLRGG* |
| 6KR+K48 Ub | MQIFVRTLTGRTITLEVEPSDTIENVRARIQDREGIPPDQQRLIFAGKQLEDGSAWSHPQFEKSATLSDYNIQRESTLHLVLRLRGG* |
| 6KR+K63 Ub | MQIFVRTLTGRTITLEVEPSDTIENVRARIQDREGIPPDQQRLIFAGRQLEDGSAWSHPQFEKSATLSDYNIQKESTLHLVLRLRGG* |
| 5KR+K27-63 Ub | MQIFVRTLTGRTITLEVEPSDTIENVKARIQDREGIPPDQQRLIFAGRQLEDGSAWSHPQFEKSATLSDYNIQKESTLHLVLRLRGG* |

*Ubiquitin lysines are shown in red. Strep tag sequence in highlighted in green
